# Measles resurgence in the United States: epidemiological and clinical observations from 2025

**DOI:** 10.1097/JS9.0000000000003499

**Published:** 2025-09-09

**Authors:** Alexander Johnson, Srilakshmi Penmetsa, Abubakar Nazir

**Affiliations:** a The Jewish Hospital – Mercy Health, Cincinnati, OH, USA; b Oli Health Magazine Organization, Research and Education, Kigali, Rwanda

*Dear Editor,*

Measles cases in the United States have surged in 2025, with 1319 confirmed cases as of 22 July, reported across 40 jurisdictions, compared to just 285 cases in the entirety of 2024, a more than 4.5-fold increase^[1,2]^. There have been 29 confirmed outbreaks this year, accounting for 87% of all cases (1154/1319), compared to 16 outbreaks in 2024, which represented 69% of total cases^[3,4]^. The purpose of this editorial is to highlight the alarming resurgence of measles in the United States, contextualize it within historical trends, and examine the public health, societal, and policy implications of this surge. In line with current publishing standards on responsible AI usage and reporting, this study was conducted and reported in compliance with the TITAN 2025 Guidelines on transparent integration of AI in academic work^[5]^.

The United States is experiencing its largest measles resurgence since 1992, when over 2100 cases occurred. Between 1 January and 20 March 2025, the WHO reported 378 cases across 17 states, including 2 confirmed deaths and a 17% hospitalization rate. That early window alone surpassed many previous full-year totals^[2,3]^. In Texas and New Mexico, key hotspots, 259 measles cases were reported by mid-March in Texas (99% unvaccinated), involving 34 hospitalizations and the first death of a child in a decade. New Mexico reported 35 cases and 1 adult death, all in unvaccinated individuals^[2,3]^.

By mid-May, Center for Disease Control (CDC) data confirmed 1024 cases across 31 states, with 92% of cases tied to 14 outbreaks and 13% requiring hospitalization (128 of 1024)^[2,3]^. Among hospitalized children under 5 years, the rate was 23%. All three total deaths by that point were in unvaccinated individuals^[2,3]^ (Table 1). As of late May, more than 729 cases in West Texas alone were associated with hospitalizations (at least 94 across Texas) and 2 child fatalities; New Mexico had 79 cases, including 1 death. Outbreaks also spread to Kansas (58 cases) and Ohio (41)^[2,4]^.

**Table 1:** Summary of key statistics on the 2025 US measles resurgence

Category	Data/statistic
Total US cases (2025 YTD)	1,319 cases (as of 22 July 2025)
Total cases in 2024	285 cases
Increase vs. 2024	4.5 × higher
Number of states affected	39–40 states/jurisdictions
Number of outbreaks	29 outbreaks in 2025 (87% of all cases)
Outbreaks in 2024	16 outbreaks (69% of 2024 cases)
Hospitalization rate	11–13% overall; ~23% for children <5 years
Deaths (2025)	3 confirmed deaths (all unvaccinated)
Vaccination status	96–97% unvaccinated or unknown status
Age distribution	30% under 5 years; 38% ages 5–19; 32% adults
Kindergarten MMR rate	92.7% (2023–2024) – below 93–95% herd immunity threshold
Basic reproduction number (R₀)	12–18
Epicenter regions	Gaines County, West Texas; spread to NM, OK, KS, IN, and OH
Hospitalizations in Texas	≥94 documented; 2 child deaths
Hospitalizations nationwide	~1 in 8 cases

Demographics show 30% of cases occurred in children under 5 years, 38% in those aged 5–19 years, 32% in adults 20+, and 96% of cases are in individuals unvaccinated or with unknown statu**s**. Only around 1% had one dose of Mumps measles Rubella (MMR) and 2% had two documented doses^[2,3]^. Case hospitalization rates also vary by age: 23% under 5 years, 9% among those 5–19, and 8% in adults 20+. Three confirmed deaths have occurred in all unvaccinated individuals and represent the first US measles mortalities in over a decade^[3,4]^.

The primary epicenter is Gaines County, West Texas, with spill-over to neighboring counties such as Terry, Dawson, Yoakum, Lubbock (which included two child deaths), Ector, Lynn, Martin, and Dallam. Combined, these counties account for over 300 cases in Texas alone, exceeding nationwide yearly totals from 2024 by March^[2,3]^. Outbreaks have now reached states as geographically diverse as Alaska, California, Florida, Georgia, Kansas, Kentucky, Marylsand, Michigan, Minnesota, New Mexico, New York, Ohio, Oklahoma, Pennsylvania, Rhode Island, Tennessee, Vermont, Washington, and others. In total, 34 states had confirmed cases by June, including Kansas, where 24 deaths among children aged 0–4 years were noted in one report, though this figure seems inconsistent with mortality totals elsewhere and may reflect reporting error or misinterpretation in the media^[2,3]^ (Table 1).

Several key factors drive this resurgence: declining MMR vaccination rates tracking at approximately 92.7% for kindergartners in 2023–2024, below the 93–95% threshold needed for herd immunity; widespread vaccine hesitancy and misinformation; and lapses in public health infrastructure, including recent US Health Services leadership changes that resulted in dismantled advisory panels and reduced vaccine funding^[3,4]^. New surveillance tools such as wastewater detection of measles virus are now providing early warnings of silent spread, adding urgency to public health efforts in states including California, Connecticut, and Maryland The implications are severe: measles remains among the most contagious diseases (*R*₀ = 12–18), and complications include pneumonia, encephalitis, and immune amnesia, which increases susceptibility to other infections^[2,3,5]^. Hospitalization rates reach nearly 1 in 20 cases, and fatality rates can be up to 1–3 per 1000 infections in unvaccinated populations. Young children and immunocompromised individuals are at the highest risk^[2,4]^ (Table 1).

To curb this threat, urgent action is required: ramping up catch-up vaccination and booster campaigns, particularly in low-coverage communities; culturally sensitive outreach involving trusted leaders; expanding wastewater and case surveillance; restoring vaccine policy and advisory infrastructure; and prioritizing science-based public messaging^[4]^. Without such a coordinated response, the US risks losing its measles elimination status and undoing decades of progress against vaccine-preventable diseases.

The US measles outbreak of 2025, with 1319 cases so far, three deaths, and widespread geographic spread, underscores the fragility of herd immunity and the consequences of declining trust in vaccines and public health institutions^[2-4]^ (Table 1). Only a comprehensive, multi-pronged response can prevent further escalation of this preventable crisis.

## Data Availability

Not applicable.
